# Correlation between Cardiovascular Autonomic and Pulmonary Ventilation Functions in Myasthenia Gravis Patients

**DOI:** 10.3390/arm91060040

**Published:** 2023-12-01

**Authors:** Monika Zawadka-Kunikowska, Łukasz Rzepiński, Mirosława Cieślicka, Joanna Fanslau, Jacek J. Klawe, Małgorzata Tafil-Klawe

**Affiliations:** 1 Department of Human Physiology, Nicolaus Copernicus University in Torun, Ludwik Rydygier Collegium Medicum in Bydgoszcz, Karłowicza 24, 85-092 Bydgoszcz, Poland; m.cieslicka@cm.umk.pl (M.C.); joannaf@cm.umk.pl (J.F.); malg@cm.umk.pl (M.T.-K.); 2 Sanitas—Neurology Outpatient Clinic, Dworcowa 110, 85-010 Bydgoszcz, Poland; luk.rzepinski@gmail.com; 3 Department of Neurology, 10th Military Research Hospital and Polyclinic, 85-681 Bydgoszcz, Poland; 4 Department of Hygiene, Epidemiology, Ergonomy and Postgraduate Education, Nicolaus Copernicus University in Torun, Ludwik Rydygier Collegium Medicum in Bydgoszcz, M. Sklodowskiej-Curie 9, 85-094 Bydgoszcz, Poland; jklawe@cm.umk.pl

**Keywords:** myasthenia gravis, heart rate variability, autonomic dysfunction, deep breathing test, pulmonary function, baroreflex sensitivity

## Abstract

**Highlights:**

**What are the main findings?**

- A lower baroreflex sensitivity in the MG group was associated with worse clinical disease outcomes and reduced pulmonary function.
- Age, forced vital capacity, and total lung capacity could predict the heart rate response during deep breathing.

**What is the implication of the main finding?**

- There is a significant relationship between reduced pulmonary ventilation function or respiratory mechanics and cardiovascular autonomic parameters in MG patients.
- Future studies should prioritize investigating how respiratory and autonomic function testing interact, alongside exploring the impact of pulmonary rehabilitation.

**Abstract:**

This study aimed to investigate the relationship between pulmonary function and cardiac autonomic function parameters in clinically stable myasthenia gravis (MG) patients. A total of 22 MG patients and 22 healthy controls (HCs) were evaluated. Pulmonary function test parameters, heart rate variability (HRV), baroreflex sensitivity (BRS), and cardiovascular autonomic function test parameters (the Valsalva ratio, expiration/inspiration (E/I) ratio) were assessed. Compared with the HCs, the patients demonstrated a similar diffusion capacity for carbon monoxide (D_LCO_); a lower forced vital capacity (FVC%_pred_); a lower forced expiratory volume in 1 s (FEV1%_pred_); lower BRS and HRV, including high-frequency and total power spectral density; and a higher percentage of abnormal cardiovagal function test results (*p* < 0.05). A lower BRS in the patient group was associated with worse clinical disease outcomes and reduced pulmonary function (D_LCO_%_pred_, R = 0.59; TLC%_pred_, R = 0.48). Age, forced vital capacity, and total lung capacity predicted the E/I ratio (R^2^ values ranging from 0.48 to 0.49). Our study demonstrated a significant relationship between a reduced pulmonary ventilation function and respiratory mechanics with cardiovascular autonomic parameters, including the E/I ratio, BRS, and HRV measures at rest, as shown in the MG group. Future studies should focus on the interplay between respiratory and autonomic function testing, as well as pulmonary rehabilitation, to mitigate cardiovascular risk in these patients.

## 1. Introduction

Myasthenia gravis (MG) is a rare autoimmune disease characterized by skeletal muscle weakness and fatigue; it involves the production of autoantibodies directed against the postsynaptic nicotinic acetylcholine receptor (AChR) and muscle-specific receptor tyrosine kinase (Musk) [1]. Moreover, the matrix metalloproteinases (MMPs), a family of zinc-dependent extracellular matrix (ECM) remodeling endopeptidases, have recently been suggested as potential biomarkers in MG [2]. The most serious complication of MG is respiratory dysfunction, which affects approximately 30–40% of adults throughout the course of the disease. In addition, myasthenic crisis and respiratory comorbidities are associated with an increased mortality rate in MG [3,4]. Ventilatory muscle weakness significantly contributes to respiratory muscle involvement in MG patients, who have reduced spirometric values as measured via the forced expiratory volume in the first second (FEV_1_) and forced vital capacity (FVC) [5,6]. Lung function assessment is one of the important evaluation measures to determine acute exacerbation [7], as well as chronic stable disease [8]. It has been reported that even well-regulated patients may exhibit ventilatory impairments defined by mild restrictive or mixed restrictive/obstructive patterns [8,9]. In addition, about 11–21% of patients with MG may develop chronic pulmonary diseases, including asthma [3]. Although the etiology of MG remains unknown, it has been determined that inflammation and the modulation of the immune response by cytokines play a role [10,11].

Physiological evidence suggests that an excessive or persistent chronic inflammatory state has also been shown to affect the autonomic nervous system (ANS) [12]. Interactions between cross-reactivity and autonomic imbalance, characterized by decreased vagal tone and overstimulation of the sympathetic nervous system, are also known as key players in the cardiac involvement in MG [13]. Assessing heart–lung interactions in MG may be important since respiratory and cardiovascular functions are intimately linked through the ANS [14]. Furthermore, the respiratory pattern modulates both baroreflex function and the sympathetic and parasympathetic neural drive to the heart, and this should be considered in the interpretation of heart rate variability (HRV) [14,15,16]. Several cardiac manifestations have been reported in association with MG, including left cardiac autonomic imbalance, ventricular abnormalities, myocarditis, cardiac arrhythmia, and heart failure [12,17,18,19]. A recent meta-analysis showed that as a group, MG patients have an altered cardiac autonomic function, including a decreased parasympathetic function, lower baroreflex sensitivity (BRS), and higher sympathovagal balance at rest and during orthostatic challenges [20]. Furthermore, reduced BRS and decreased lung function have been linked to an increased risk of cardiovascular mortality [21,22]. The associations between cardiac autonomic regulation and pulmonary function have been investigated in respiratory and metabolic diseases such as chronic obstructive pulmonary disease [23,24], diabetes [25], and morbid obesity [26], as well as in patients in the acute and chronic phases of COVID-19 [27]. However, at present, the correlation between pulmonary function and cardiovascular autonomic regulation, including BRS, HRV, and autonomic function test parameters, has not been assessed in the MG population. Thus, the objective of this study was to determine the relationship between pulmonary ventilation function and cardiovascular autonomic parameters in clinically stable MG patients.

## 2. Material and Methods

This was a case–control study conducted between December 2021 and May 2023. Twenty-two patients diagnosed with MG were included in this study, which was carried out with twenty-two age-matched healthy subjects (HCs) serving as the controls. The study was approved by the Bioethical Committee of the Collegium Medicum in Bydgoszcz, Nicolaus Copernicus University in Torun (KB 747/2017), and informed consent was obtained from all participants.

The diagnosis of MG was based on clinical presentation (fluctuating weakness of ocular and/or extraocular muscles) and at least one of the following criteria: positive AChR autoantibodies (AChR-Abs) or MuSK antibodies (MuSK-Abs), electrophysiological findings (repetitive stimulation and/or single-fiber electromyography), or a clinical response to cholinesterase inhibitors. The inclusion criteria for the MG patients were as follows: an age greater than 18 years, a confirmed diagnosis of MG, and a stable course of the disease for a minimum period of three months, defined as no clinical deterioration resulting in an increase in the MGFA classification by at least one class [28]. The exclusion criteria for MG patients and controls included: an acute respiratory infection or MG crisis in the previous 3 months, a history of recent surgical procedures in the chest area, diagnosed concomitant respiratory disease, a history of severe heart disease, atrial fibrillation, evidence of facial weakness hindering an accurate spirometry examination, cognitive/psychiatric comorbidities impeding the proper interpretation of instructions, and treatment with beta-blockers and antihypertensive drugs. HCs were recruited from the local community (northern Poland).

In this study, the demographic characteristics, detailed medical history, laboratory findings, and symptoms of all enrolled patients with MG were collected from their medical records (Table 1). Patients were clinically classified into classes I–V by a neurologist according to the Myasthenia Gravis Foundation of America (MGFA) classification: pure ocular MG (class I), mild generalized MG (class II), moderate generalized MG (class III), severe generalized MG (class IV), and intubation/myasthenic crisis (class V) [29] (Table 1). Serum levels of AChR antibodies were detected with an enzyme-linked immunosorbent assay (ELISA). IgG4 antibodies against MuSK were measured via an ELISA in subjects lacking anti-AChR antibodies. The thymic pathology was assessed in accordance with the CT imaging and available histology findings. In all patients, the cardiac autonomic and pulmonary functions were assessed while receiving treatment for MG (Table 1).

### 2.1. Pulmonary Function Tests (PFTs)

We performed pulmonary function tests (PFTs) according to the American Thoracic Society/European Respiratory Society criteria using the Ganshorn PowerCube Body+ and Diffusion+ (Schiller Group, Niederlauer, Germany) [30,31]. PowerCube body plethysmography can be used to measure the spirometry and flow volume characteristic values. The tests were conducted in the sitting position, with participants wearing a nose clip and breathing through a mouthpiece. PFT parameters were expressed as both absolute values and as percent predicted values, which were calculated using the Global Lung Function Initiative (GLI-2012) reference equations The final measurement included the determination of ventilation, diffusion function, and static lung volumes via whole-body plethysmography. Spirometry was performed without a bronchodilator. The interpretation of diffusing capacity values was derived from the official technical standards established by the ERS/ATS [32].

The lower limit of normal (LLN) was defined as the 5th percentile, corresponding to a Z-score of −1.645. We utilized the recommendations of the ATS/ERS Task Force to define airway obstruction and restriction. An obstructive ventilatory impairment was characterized as an FEV_1_/FVC below the LLN, which was determined to be in the 5th percentile of the normal population. A restrictive ventilatory impairment was identified as a reduction in the TLC below the LLN, which was also set at the 5th percentile [32]. A professional technician reviewed the content to exclude obvious errors due to poor patient cooperation. The patients were asked to perform at least three acceptable tests, with a maximum of 5 test, and the best values were recorded.

The following parameters were derived: vital capacity as a percentage of expected value (VC), inspiratory volume as a percentage of expected value (IC), total lung capacity as a percentage of expected value (TLC), residual volume as a percentage of expected value (RV), ratio of residual volume to total lung capacity (RV/TLC), forced vital capacity as a percentage of expected value (FVC), forced expiratory volume in 1 s as a percentage of expected value (FEV_1_), ratio of forced expiratory volume in 1 s to forced vital capacity (FEV_1_/FVC), ratio of forced expiratory volume in 1 s to vital capacity (FEV_1_/VC_max_), peak inspiratory flow (PIF), peak expiratory flow as a percentage of expected value (PEF), mid-expiratory flow 50 (MEF50), mid-inspiratory flow 50 (MIF50), specific airway resistance (sRAW), total airway resistance (Rtot), specific total airway resistance (sRtot), diffusing capacity for carbon monoxide (D_LCO_), and coefficient of transfer factor for CO (K_CO_).

### 2.2. Cardiovascular Autonomic Testing

The autonomic function was measured using TFM software version 2.3.20.20 (TFM, CNSystems Medizintechnik, Graz, Austria). Measurements were carried out at the same time of day, between 8 and 12 a.m., in a quiet, darkened, comfortable room where the temperature was maintained at around 22 ± 1 °C. All examinations were performed by the same investigator. All participants were instructed to avoid alcohol and caffeine consumption, smoking, and intensive exercise for at least 12 h before testing [33,34].

Heart rate (HR) measurements were obtained from a standard ECG. The respiration rate was calculated using thoracic impedance [31]. The ANS function was evaluated via a power spectral analysis of the short-term heart rate (HRV) using the frequency-domain method. For all subjects, the recordings were conducted in a supine position for a minimum of 10 min, following a preceding 5–10 min stabilization period. HRV was obtained from the beat-to-beat data that were obtained using an adaptive autoregressive (AAR) model, as described by Bianchi et al. [35]. For the frequency-domain HRV, the following parameters were measured: power in the low-frequency band (LF, 0.04–0.15 Hz), reflecting both sympathetic and parasympathetic activity; power in the high-frequency band (HF, 0.15–0.40 Hz), reflecting parasympathetic and respiratory activity; total power spectral density (PSD), representing the total variability and global index of HRV; and LF/HF-RRI and LF/HF ratios, reflecting sympathovagal balance. For analysis, results were expressed in absolute terms (ms^2^) [35,36]. BRS at rest was determined by calculating the slope of the regression between spontaneous occurring sequences of blood pressure changes and concurrent variations in the RR interval. Deep breathing and the Valsalva maneuver were used as indicators of parasympathetic function. We calculated the Valsalva ratios (the maximum/minimum ratio of the RR interval during a Valsalva maneuver) and expiration/inspiration ratios (the ratio of the longest R–R interval during expiration to the shortest R–R interval during slow, deep breathing at 6 cycles per minute) [33].

### 2.3. Statistical Analysis

In our study, we extracted HRV data from the TFM program and transferred it to Microsoft Excel for subsequent analysis. Subsequently, all data were imported into Statistica version 13. To eliminate outliers/artifacts, Grubbs’ test was applied to filter out all HRV beat-to-beat data [37]. Data were expressed as number (*n*), percentage (%), mean ± SD, or median interquartile range (IQR) based on variable distribution. Differences in the distribution of qualitative variables were determined with the chi-square test or Fisher’s exact test. The parametric Student *t*-test and the nonparametric Mann–Whitney test were used to compare intergroup differences. Spearman’s correlation was employed to examine the associations between pulmonary ventilation parameters, clinical disease outcome markers, and cardiovascular autonomic indices. Multiple regression analysis with two predictors was employed to explain the relationship between cardiovascular autonomic indices and the independent variables (demographic and pulmonary). The results were determined at a 95% confidence interval, and the level of significance for all tests was set at *p* < 0.05.

## 3. Results

### 3.1. Demographic and Clinical Data

We included 22 patients with clinically stable MG: the mean age was 42.45 ± 7.19 years and 17 (72.27%) were female. The distribution across the MGFA classes was: 4.5%, 31.0%, 40.9%, and 54.5% for classes I–IIIa, respectively. The mean BMI was 25.42 ± 4.34 kg/m^2^. The estimated mean disease duration for the MG group was 6.93 ± 8.02. Among the 22 HCs, the mean age was 37.32 ± 12.17 and 17 were female (77.27%). The mean BMI was 23.66 ± 3.81 kg/m^2^. There were no statistically significant differences between the MG patients and HCs regarding age, gender, and BMI (*p* > 0.05) (Table 1). Cholinesterase inhibitors were used by 95% of the patients (pyridostigmine, mean dose 240 mg/day), whereas 59% used corticosteroids (prednisone, mean dose 30 mg/day) and 36.4% used an immunosuppressive therapy (6/27.3% azathioprine, mean dose 150 mg/day and 2/9.1% mycophenolate mofetil, mean dose 1000 mg/day). The full demographics and clinical characteristics of the MG patients and HCs are presented in Table 1.

### 3.2. Pulmonary Function Tests: MG Patients vs. HCs

**Figure 1:**
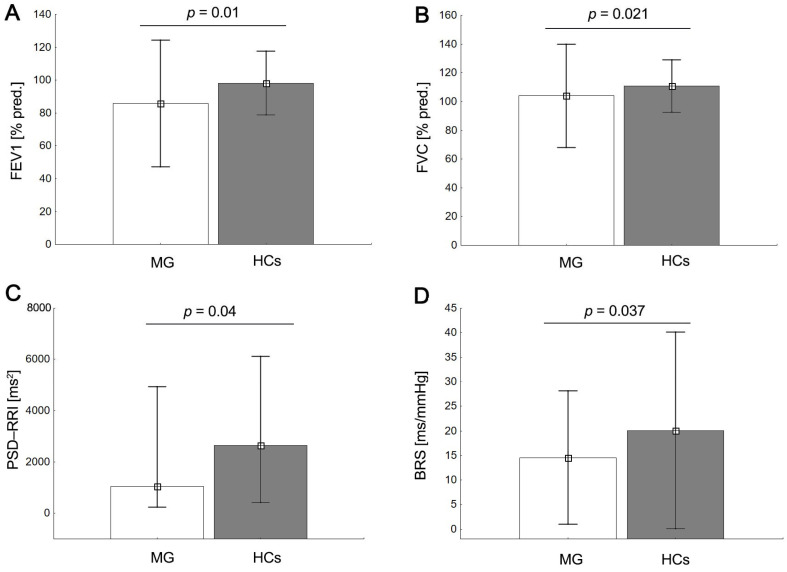
Mean ± SD values of pulmonary and autonomic measurements; forced expiratory volume in 1 s as a percentage of expected value (FEV1(%pred)) (**A**), forced vital capacity (FVC) (**B**), power spectral density of HRV (PSD-RRI) (**C**), and baroreflex sensitivity (BRS) (**D**) in the MG group compared with HCs.

The pulmonary function parameters are summarized in Table 2. Several tests could not be performed in some patients because of neuromuscular deficits (spirometry and body plethysmography: 22 MG patients vs. 22 HCs; diffusion capacity: 15 MG patients vs. 22 HCs). The mean levels of pulmonary ventilation and respiratory mechanics were within the normal levels for all groups. Among the MG group, one patient (a smoker) presented an obstructive pattern (lower FEV_1_, normal FVC, lower FEV_1_/FVC, and normal TLC), two presented a muscle weakness pattern (lower FEV_1_, lower FVC, normal FEV_1_/FVC, and normal TLC), and a third presented a mixed disorder based on spirometry and lung volume parameters [30]. Finally, 4 out of 15 (26.66%) patients with MG had pulmonary diffusion dysfunction, with a DLCO below the LLN. Overall, significant differences were observed in the mean values of VC_max_, FVC, FVC (%pred), FEV_1_, FEV_1_ (%pred), and RV/TLC across the MG and HC groups (*p* < 0.05) but no statistically significant difference was found for the diffusion capacity parameters, as shown in Table 2 and Figure 1. Pulmonary hyperinflation (RV/TLC > LLN) was found in 13 (60.0%) patients with MG but was not present in the HC group.

### 3.3. Autonomic Function

All participants in the study exhibited a normal sinus rhythm. At rest, values for RRI and HR were similar in both groups (Table 3). In addition, the MG patients, compared with the HCs, were characterized by a significantly higher percentage of abnormal cardiovagal function test results and lower values of BRS (*p* = 0.037) and HRV indices (HF-RRI, *p*= 0.038; PSD-RRI, *p* = 0.040). In contrast, despite the lower values of the LF-RRI and the higher sympathovagal balance ratio (LF/HF, LF/HF-RRI) in the MG subjects, no significant differences were observed between the groups (*p* > 0.05; Table 3, Figure 1). When recording the HRV at rest, the MG patients and HCs exhibited a similar breathing rate.

### 3.4. Relationship between Cardiovascular Autonomic, Pulmonary Function Parameters, and Clinical Disease Outcomes

**Figure 2:**
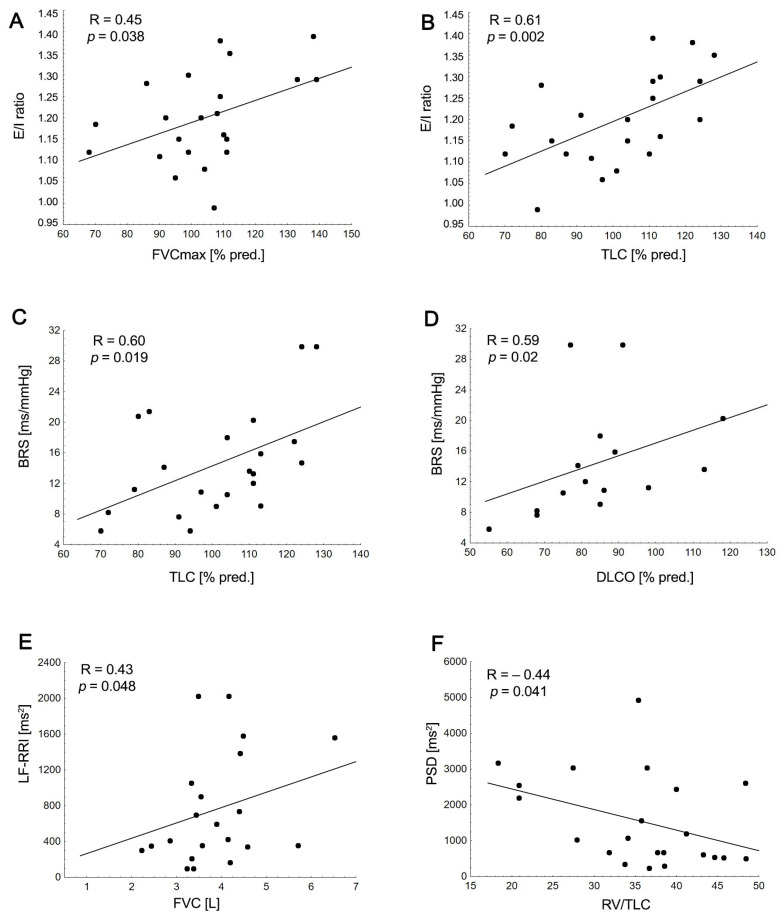
Associations between the high frequency of the E/I ratio with HRV (HF-RRI) with force vital capacity (FVC) (%pred) (**A**); the E/I ratio with total lung capacity (TLC (%pred)) (**B**); baroreflex sensitivity (BRS) with total lung capacity (TLC (%pred)) (**C**); baroreflex sensitivity (BRS) with diffusing capacity for carbon monoxide (D_LCO_ (%pred)) (**D**); low frequency of HRV (LF-RRI) with force vital capacity (FVC) (L) (**E**); power spectral density of HRV (PSD-RRI) and ratio of residual volume to total lung capacity (RV/TLC) (**F**).

In the MG group, the HF-RRI and PSD-RRI were positively related to PIF (R = 0.44, *p* = 0.04; R = 0.45, *p* = 0.034) and MIF50 (R = 0.48, *p* = 0.023; R = 0.48, *p* = 0.025), respectively. Similarly, the LF-RRI was positively associated with FVC (Figure 2). The RV/TLC ratio was inversely related to LF-RRI (R = −0.47, *p* = 0.028) and PSD-RRI (Figure 2) and was positively associated with disease severity (MGFA score: R = 0.44, *p* = 0.041). Interestingly, the sympathovagal balance (LF/HF, LF/HR-RRI) was inversely related to TLC (%pred) (R = −0.43, *p* = 0.045; R = −0.45, *p* = 0.035) and RV (%pred) (R = −0.51, *p* = 0.016; R = −0.44, *p* = 0.039). Airway resistance parameters (sRAW (%pred)) were inversely correlated with the sympathovagal balance ratio (LF/HF: R = −0.44, *p* = 0.039; LF/HF-RRI: R = −0.56, *p* = 0.007) and positively associated with the HF-RRI (R = 0.46, *p* = 0.003). BRS was inversely correlated with disease duration (R = −0.47, *p* = 0.028), disease severity (MGFA score: R = −0.45, *p* = 0.034), D_LCO_ (%pred) and TLC (%pred) (Figure 2).

Moreover, the MGFA score was inversely associated with the pulmonary diffusion capacity parameters (DLCO (%pred): R = −0.71, *p* = 0.003; KCO (%pred): R = −0.51, *p* = 0.05), BRS, and PSD-RRI (R = −0.45, *p* = 0.034). The disease duration correlated only with the PSD-RRI (R = −0.45, *p* = 0.036) and BRS. Age was inversely correlated with the E/I ratio (R = −0.49, *p* = 0.02) and PSD-RRI (R = −0.43, *p* = 0.045) and was positively associated with the expiratory flow parameters (MEF50 (%pred): R = 0.43, *p* = 0.045; MEF20 (%pred): R = 0.54, *p* = 0.009).

Regarding cardiovascular autonomic function tests, only the E/I ratio was positively correlated with FVC (%pred) and TLC (%pred), Figure 2.

In the multivariable regression model, age (b = −0.64, *p* < 0.001) and FVC (%pred) (b = 0.55, *p* = 0.002) predicted the E/I ratio (R^2^ = 0.53, *p* = 0.002), explaining 53% of the variance. Similarly, age (b = −0.43; *p* = 0.018) and TLC (%pred) (b = 0.47; *p* = 0.011) were identified as independent predictors of the E/I ratio (R^2^ = 0.44, *p* = 0.014), explaining 44% of the variance.

## 4. Discussion

In this preliminary study involving individuals with MG, we found that PFT estimates representing pulmonary ventilation function and respiratory mechanics were associated with cardiovascular autonomic parameters, including the E/I ratio, BRS, and HRV measures at rest. Furthermore, in the MG group, we demonstrated that age, forced vital capacity, and total lung capacity predicted the HR response during the DBT (E/I ratio). Moreover, lower baroreflex sensitivity in the patient group was associated with worse clinical disease outcomes and reduced pulmonary function (D_LCO_ and TLC).

In line with previous studies, we observed that MG patients, when compared with controls, exhibited a greater similar diffusion capacity function and lower FEV_1_ and FVC values, all of which generally fell within normal limits. However, 26% (4/15) of MG patients exhibited impaired gas exchange (D_LCO_ < LLN) and 18.2% (4/22) demonstrated ventilatory impairments such as airflow obstruction or restriction. These alterations reflect a myasthenic breathing pattern resulting from a reduced volume and ventilatory muscle weakness [5,6,38]. We also found that a greater disease severity was associated with lower spirometry measures, a worse diffusion capacity (D_LCO_, K_CO_), and the occurrence of functional pulmonary hyperinflation. In this context, our findings highlight the significance of subclinical deterioration in respiratory mechanics due to poor respiratory muscle strength, along with a concomitant reduction in the functional lung surface area. This, in turn, impacts gas exchange in clinically stable MG patients [39]. A lower FEV_1_ and D_LCO_ have recently emerged as negative prognostic factors based on data from a retrospective study that investigated independent risk factors for postoperative myasthenic crises in MG patients. Jiao et al. studied 564 MG patients who underwent a standard expanded resection of thymoma and found that the independent risk factors for an MG crisis were a low maximum ventilation volume value and low FEV_1_ and D_LCO_ values. They concluded that the risk of an MG crisis increases when MG patients are susceptible to respiratory muscle weakness and when there is an alteration in the diffusion capacity function [7]. Similarly, a recent meta-analysis of 18 studies showed that the pulmonary diffusing capacity was the most common impaired lung function in patients who recovered from COVID-19 [40].

In terms of cardiac autonomic function, MG patients exhibited a decreased HRV (HF and PSD), a lower BRS, a higher prevalence of abnormal cardiovagal function test results (the Valsalva ratio or E/I ratio), and a tendency toward a slightly higher but not statistically significant sympathovagal balance compared with that in HCs. These results partially corroborate the findings from our recent meta-analysis [20] involving 301 MG patients and 454 healthy controls, in which we confirmed a decreased parasympathetic function, reduced BRS, and increased sympathovagal balance at rest and during orthostatic challenges in patients with MG. However, some differences in our study may result from variations in sample size and disease severity.

Prior research suggests a potential link between pulmonary ventilation and cardiovascular autonomic function, including an HR response to the deep breathing test [22], BRS [41], and HRV measures [23,25,26,27]; however, this study is the first to confirm this association in clinically stable MG patients.

In the present study, we found that age, FVC (%_pred_), and TLC (%_pred_) were independent predictors (with R^2^ values ranging from 0.48 to 0.49) of the lower HR response to deep breathing (E/I ratio) in MG patients. These findings may partly explain the high heterogeneity in MG studies that assess the E/I ratio [20]. Our results are consistent with those of previous studies that link diminished cardiovagal tone and/or increased sympathetic activity at the sinus node with reduced pulmonary function [24]. Accordingly, Reis et al. compared 10 patients with COPD to 9 HCs and found positive correlations between the maximal inspiratory pressure and the results of the deep breathing test, expressed as the inspiratory–expiratory difference (I–E). The heart–lung interaction in terms of respiratory sinus arrhythmia using HRV measures was also observed in our study [42]. Specifically, in our study, the LF of HRV, representing both the sympathetic and parasympathetic branches of the ANS, decreased with FVC. These findings are consistent with those of Sperandio et al., who studied 119 HCs and found a positive correlation between the spirometry indices (FEV_1_, FVC) and linear and nonlinear indices of HRV, including in the low-frequency and long-term standard deviation of the Poincaré plot (SD2) [43]. In line with these findings, Bianchim et al., in a sample of 119 adults, also confirmed a positive relationship between FEV_1_ and FVC and parasympathetic activity and overall autonomic variability, respectively. They concluded that pulmonary function is influenced by the autonomic control of cardiovascular function independently of the main confounders (age, sex, smoking, physical inactivity, and cardiovascular risk) [44].

The FVC and FEV_1_ appeared to be strong predictors of survival in the general adult population [21]. Although we recruited only clinically stable MG patients, four (18%) of them exhibited a lower FEV_1_ and two had a lower ratio of FEV_1_/FVC below the LLN. A large prospective study with 25,639 participants demonstrated that participants with poorer lung function (lower FEV_1_) are at greater risk of total and cardiovascular mortality, even in the absence of cardiovascular disease [21]. This appears to be particularly important in elderly MG patients, who may experience a high total cardiopulmonary load [3]. In our study, a lower HRV (HF, PSD) was negatively associated with peak inspiratory flow parameters, potentially resulting in inspiratory flow limitations, increased inspiratory effort due to high intrathoracic pressure, and a higher amount of work required for breathing [3,14]. Previous studies suggest an inverse relationship between lung hyperinflation and cardiac function, which includes the cardiac preload, BRS, and HRV. Consistent with these findings, 62% of the patients exhibited functional pulmonary hyperinflation with higher RV/TLC ratios, which correlated with a depressed HRV (LF, PSD). This association could be attributed to increased sympathetic activation [45]. Accordingly, Mayr et al., in patients with COPD, revealed that hyperinflation may alter vagal activity, which is represented by a decrease in BRS, through alterations to the mechanosensitive afferent vagal nerve function in the lungs [41].

Interestingly, in our study, compared with HCs, MG patients showed a lower BRS, which was inversely correlated with a lower diffusion function (D_LCO_) and total lung capacity. Multiple possible mechanisms could explain the relationship between a lower BRS and reduced pulmonary function. Firstly, both the reduced diffusion capacity function and diminished BRS were associated with worse disease outcomes, suggesting a possible parallel progression for impaired gas exchange and impaired cardiovascular control. Secondly, although MG patients showed a tendency toward having slightly higher, but not statistically significant, breathing rates, there exists a reciprocal interaction between the reduction in BRS, sympathetic overactivity, and increased respiratory ventilation. Indeed, as MG induces an altered ventilatory pattern, respiratory muscle weakness, and chest tightness, it may augment rapid shallow breathing and thereby result in a higher degree of sympathetic activation and parasympathetic withdrawal [15]. The purpose of rapid shallow breathing is to alleviate the perceived respiratory effort; however, it ultimately results in hypercapnia (chemoreflex response) in these patients [15,16]. Thirdly, increased sympathetic activation diminishes HRV and BRS, potentially resulting in low-grade systemic inflammation, which is another key player in respiratory failure [10,12]. A previous study provided evidence of systemic inflammation in patients with MG, expressed as abnormally elevated levels of immune-related markers in blood cell counts [8]. A relationship between autonomic dysfunction, low-grade inflammation, and a worse pulmonary function was also described in COPD patients [23]. Fourthly, altered cardiovascular autonomic function may induce functional alterations in the regulation of pulmonary microvascular tone and the distribution of pulmonary blood flow, ultimately resulting in a decrease in pulmonary capillary blood volume, as described in diabetic and fibromyalgia patients [46].

Finally, in patients with MG, the airways can be secondarily influenced by the interplay between alterations in bronchial smooth muscle tone and, primarily, the loss of lung elastic recoil as the lung volume declines. Though, it should be noted that we included clinically stable, treated MG patients in our analysis, which may partly explain the potential similar values between the groups regarding airway resistance. Previous experimental studies suggest that the activation of β2-adrenergic receptors in airway smooth muscle causes bronchodilation and decreases airway resistance [47]. In line with these findings, in our study, a lower sRAW correlated with a higher sympathovagal balance at rest. In contrast, Horwath et al. found that, under resting conditions, the vagal control of bronchial tone (RAW) and the heart period were not related in HCs [48]. Taken together, these findings may indicate that impaired pulmonary function might be one aspect influencing functional autonomic imbalance and ANS dysregulation in MG patients. Moreover, the incorporation of both respiratory and ANS assessments could potentially enhance health outcomes.

There were a few limitations to our study. First, the sample size and the study power were limited by the number of patients. Second, medication including acetylcholinesterase inhibitors and immunosuppressive agents may mediate the observed effects on pulmonary and cardiac autonomic function, as well as affect clinical outcomes. Previous studies have shown that the prolonged use of pyridostigmine enhances cardiac autonomic control, including improvements in vagal tone, BRS, and HRV. In our previous study, we established the presence of diminished cardiovagal tone in MG patients, even as the acetylcholinesterase inhibitors led to an enhancement in vagal tone [17,49]. Considering the evaluation of HRV, future studies could focus on establishing stable therapies for MG before conducting the assessment, maintaining no dose changes for at least 3 months (e.g., corticosteroids). Thirdly, only some of the patients had all pulmonary function tests available for analysis, which might cause potential bias. Fourthly, we did not take changes in hemoglobin into account when interpreting the D_LCO_ results, nor did we account for measures of inspiratory/expiratory muscle strength or the level of physical activity, which can influence HRV. Notable strengths of our study include a well-characterized cohort of patients and the use of validated methods that simultaneously assess autonomic and pulmonary function.

## 5. Conclusions

Our study demonstrated a significant relationship between a reduced pulmonary ventilation function or respiratory mechanics and cardiovascular autonomic parameters, which included the E/I ratio, BRS, and HRV measures at rest, as shown in the MG group. A lower baroreflex sensitivity in the patient group was associated with worse clinical disease outcomes and reduced pulmonary function. Furthermore, age, forced vital capacity, and total lung capacity could predict the HR response during the DBT. Taken together, given that MG patients have an altered cardiac autonomic function, future studies should focus on the interplay between respiratory and autonomic function testing, as well as pulmonary rehabilitation, to mitigate cardiovascular risk in these patients.

## Figures and Tables

**Table 1:** Demographic and clinical data of the study participants.

	HCs	Total MG	*p*-Value
Number of subjects	22	22	
Sex, female n (%)	16 (77.73)	17 (77.27)	0.727
BMI, mean (kg/m^2^)	23.66 ± 3.82	25.42 ± 4.34	0.160
Age, mean (years)	37.32 ± 12.17	42.45 ± 7.19	0.096
Age at first manifestation, mean (years)		35.32 ± 9.42	
Disease duration (years), mean (range)		6.93 ± 8.02	
Seropositivity to AChR antibodies, *n* (%)		9 (40.9)	
Seropositivity to MuSK antibodies, *n* (%)		4 (18.2)	
Seropositivity to AChR and MuSK, antibodies, *n* (%)		1 (4.54)	
Type of MG, *n* (%)			
Ocular		1 (4.5)	
Generalized		21 (95.5)	
Thymectomy, *n* (%)		7 (22)	
Severity of disease at the moment of testing (MGFA, number, %)			
Class 0		0 (0)	
Class I (ocular)		1 (4.5)	
Class IIa		9 (40.9)	
Class IIIa		12 (54.5)	
Histology changes, *n* (%)			
Thymic pathology		14 (63.6)	
Thymoma		1 (4.5)	
Unknown		1 (4.5)	
Type of treatment, *n* (%)			
Use of cholinesterase inhibitors		21 (95.5)	
Use of corticosteroids		13 (59.1)	
Use of immunosuppressants		8 (36.4)	

**Table 2:** Median [Q1–Q3] PFT parameters for mild MG patients, moderate MG patients, and HCs.

	MG		HCs		*p*
Pulmonary Volume	Mean	Median [Q1–Q3]	Mean	Median [Q1–Q3]	
VC (L)	3.67	3.60 [2.91–4.22]	4.28	4.22 [3.59–4.70]	0.037
VC (%pred)	94.12	94.50 [83.00–110.00]	105.95	107.00 [99.00–115.0]	0.059
IC (L)	2.65	2.46 [2.01–3.02]	3.01	2.80 [2.37–3.55]	0.192
IC (%pred)	101.77	96.50 [90.00–109.00]	106.73	108.50 [98.00–115.00]	0.124
RV (L)	1.98	2.13 [1.57–2.41]	1.92	1.82 [1.48–2.07]	0.753
RV (%pred)	114.23	110.50 [99.00–132.00]	110.50	107.00 [91.00–129.00]	0.737
TLC (L)	5.59	4.96 [4.75–6.39]	6.21	5.93 [5.50–6.80]	0.139
TLC (%pred)	101.32	96.00 [91.00–103.00]	106.73	105.50 [99.00–115.00]	0.262
RV/TLC	35.70	36.53 [31.82–41.16]	30.69	29.31 [24.90–36.49]	0.093
RV/TLC (%pred)	109.64	113 [94.00–128.00]	97.59	95.00 [87.00–112.00]	0.129
Pulmonary diffusing capacity					
D_LCO_ (mmol/min/kPa)	7.90	7.14 [6.01–8.87]	8.55	8.44 [7.70–9.59]	0.252
D_LCO_ (%pred)	84.53	85.00 [75.00–91.00]	88.95	90.00 [80.00–95.00]	0.315
KCO (mmol/min/kPa/L)	1.53	1.58 [1.40–1.72]	5.28	1.59 [1.40–1.75]	0.410
K_CO_ (%pred)	91.60	98.00 [84.00–100.00]	93.05	92.00 [83.00–105.00]	0.843
Pulmonary ventilation					
FVC (L)	3.70	3.57 [3.17–4.19]	4.52	4.31 [3.97–5.29]	0.051
FVC (%pred)	104.05	91.50 [80.00–99.00]	110.86	113.00 [108.00–115.00]	0.021
FEV_1_ (L)	2.84	2.74 [2.54–3.35]	3.50	3.34 [3.03–3.90]	0.011
FEV_1_ (%pred)	85.86	90.00 [80.00–96.00]	98.23	99.00 [94.00–104.00]	0.010
FEV_1_/FVC ratio (%)	76.58	79.02 [75.64–82.01]	80.16	78.95 [76.43–86.50]	0.379
FEV_1_/FVC (%pred)	93.64	97.50 [91.00–100.00]	96.73	96.50 [93.00–100.00]	0.760
FEV_1_/VCmax	72.93	75.07 [72.93–76.95]	77.90	77.21 [73.14–81.65]	0.193
FEV_1_/VCmax%	90.27	92.00 [90.00–97.00]	95.45	95.50 [89.00–100.00]	0.302
Respiratory mechanics					
PEF (L/s)	5.68	5.67 [4.55–6.68]	6.53	6.27 [5.58–7.40]	0.163
PEF (%pred)	77.82	77.50 [67.00–88.00]	84.86	82.00 [73.00–97.00]	0.282
MEF50 (L/s)	3.47	3.34 [2.86–4.59]	4.23	3.91 [3.43–4.55]	0.055
MEF50 (%pred)	79.64	82.50 [68.00–94.00]	91.55	8.50 [73.00–105.00]	0.113
MIF50(L/s)	4.21	4.41 [3.36–5.07]	4.85	4.62 [3.98–5.39]	0.149
PIF (L/s)	4.40	4.43 [3.43–5.27]	4.92	4.72 [4.32–5.32]	0.187
sRAW (kPa × s)	0.94	0.88 [0.72–1.09]	1.04	1.09 [0.72–1.34]	0.336
sRAW (%pred)	93.50	86.50 [67.00–102.00]	102.32	107.50 [75.00–124.00]	0.296
RAWtot (kPa × s/L)	0.30	0.26 [0.21–0.34]	0.31	0.27 [0.25–0.33]	0.805
sRAWtot (kPa × s/L)	98.73	84.00 [71.00–114.00]	103.91	87.50 [82.00–109.00]	0.664

**Table 3:** Median [Q1–Q3] of HRV parameters for mild MG patients, moderate MG patients, and HCs.

	MG	HCs	*p*
Breathing rate (bpm)	17.27 [14.49–19.22]	16.21 [15.04–18.22]	0.263
HR (1/min)	65.44 [62.35–65.44]	62.64 [59.30–67.92]	0.826
LF-RRI (ms^2^)	418.19 [306.76–1138.21]	1014.71 [322.75–1539.50]	0.136
HF-RRI (ms^2^)	235.30 [73.16–493.67]	463.76 [191.16–884.74]	0.038
PSD-RRI (ms^2^)	1047.04 [530.97–2565.85]	2647.00 [810.82–3824.54]	0.040
LF/HF-RRI (1)	2.14 [1.15–4.66]	1.91 [0.99–3.35]	0.307
LF/HF (1)	1.88 [0.95–2.84]	1.05 [0.85–2.12]	0.113
E/I ratio	1.19 [1.12–1.29]	1.21 [1.17–1.36]	0.058
Valsalva ratio	1.32 [1.11–1.47]	1.32 [1.20–1.70]	0.337
BRS (ms/mmHg)	13.45 [9.06–17.99]	20.05 [12.70–27.70]	0.037
Parasympathetic dysfunction (%)	27.27	4.55	0.047

## Data Availability

The datasets generated during and/or analyzed during the current study are available from the corresponding author on reasonable request.
